# The Novel Gamma Secretase Inhibitor RO4929097 Reduces the Tumor Initiating Potential of Melanoma

**DOI:** 10.1371/journal.pone.0025264

**Published:** 2011-09-29

**Authors:** Chanh Huynh, Laura Poliseno, Miguel F. Segura, Ratna Medicherla, Adele Haimovic, Silvia Menendez, Shulian Shang, Anna Pavlick, Yongzhao Shao, Farbod Darvishian, John F. Boylan, Iman Osman, Eva Hernando

**Affiliations:** 1 Department of Medicine, New York University School of Medicine, New York, New York, United States of America; 2 Department of Dermatology, New York University School of Medicine, New York, New York, United States of America; 3 Department of Pathology, New York University School of Medicine, New York, New York, United States of America; 4 Department of Environmental Medicine, New York University School of Medicine, New York, New York, United States of America; 5 Interdisciplinary Melanoma Cooperative Group, New York University School of Medicine, New York, New York, United States of America; 6 Discovery Oncology, Hoffmann-La Roche Inc., Nutley, New Jersey, United States of America; Faculdade de Medicina, Universidade de São Paulo, Brazil

## Abstract

Several reports have demonstrated a role for aberrant NOTCH signaling in melanoma genesis and progression, prompting us to explore if targeting this pathway is a valid therapeutic approach against melanoma. We targeted NOTCH signaling using RO4929097, a novel inhibitor of gamma secretase, which is a key component of the enzymatic complex that cleaves and activates NOTCH. The effects of RO4929097 on the oncogenic and stem cell properties of a panel of melanoma cell lines were tested both *in vitro* and *in vivo*, using xenograft models. In human primary melanoma cell lines, RO4929097 decreased the levels of NOTCH transcriptional target HES1. This was accompanied by reduced proliferation and impaired ability to form colonies in soft agar and to organize in tridimensional spheres. Moreover, RO4929097 affected the growth of human primary melanoma xenograft in NOD/SCID/IL2gammaR-/- mice and inhibited subsequent tumor formation in a serial xenotransplantation model, suggesting that inhibition of NOTCH signaling suppresses the tumor initiating potential of melanoma cells. In addition, RO4929097 decreased tumor volume and blocked the invasive growth pattern of metastatic melanoma cell lines *in vivo*. Finally, increased gene expression of NOTCH signaling components correlated with shorter post recurrence survival in metastatic melanoma cases. Our data support NOTCH inhibition as a promising therapeutic strategy against melanoma.

## Introduction

The incidence of melanoma has been constantly increasing during the last decades [1]. Adjuvant therapy after complete resection is recommended for thick primary melanoma with lymph node metastases, because recurrence rates are relatively high and overall survival is poor [2]. However, IFNalpha remains the only approved adjuvant therapy, which provides a modest disease-free survival benefit [3]. Furthermore, it is especially concerning that the conventionally used drugs for metastatic melanoma include dacarbazine and IL-2, both of which cause poor (<15% of cases) and transient responses [4]. Although promising therapeutic responses have been observed in recent clinical trials using the BRAF inhibitor Vemurafenib (PLX4032) and the monoclonal antibody against CTLA-4 Ipilimumab, both recently approved by the FDA, emergence of resistance and severe side effects have already been confronted [5], [6], [7], [8], [9], [10].

The NOTCH signaling pathway, which plays a role in organogenesis and cell fate determination during embryogenesis, involves four NOTCH transmembrane receptors (NOTCH 1-4) [11]. Binding of DELTA (DLL 1/3/4) or JAGGED (JAG 1/2) ligands makes the receptors susceptible to metalloprotease- and gamma secretase-mediated proteolytic cleavage. This cleavage results in the release of NOTCH intracellular domain (N^IC^) from the plasma membrane and its translocation into the nucleus. Here, N^IC^ mediates the transcription of target genes, including basic helix-loop-helix transcription factors of the hairy and enhancer of split (HES) family and the HES-related repressor protein (HERP/HRT/HEY) family.

Aberrant NOTCH signaling has been recently linked to many malignancies including melanoma, where it plays a role in progression and possibly in development [12], [13]. Mechanistically, NOTCH signaling relies on crosstalk with the Wnt/β-catenin, the MAPK/AKT, the BRN2/MITF and the NODAL pathways in order to elicit its biological functions [12], [14], [15], [16], [17].

Aberrant NOTCH signaling has also been shown to confer stem cell-like properties in different cancer types, such as breast cancer and glioma (reviewed in ref. [18]). Identification of stem cell-like tumor initiating cells has been of major interest in melanoma. Although there is an ongoing debate about the frequency and identity of melanoma initiating cells [19], [20], [21], [22], [23], the inability to eradicate this subpopulation is thought to be a reason for the failure of current treatment strategies [24]. Therefore, NOTCH inhibition in melanoma, possibly through the targeting of tumor initiating cells, can be foreseen as a new and promising therapeutic strategy.

Essential to NOTCH signaling is the catalytic cleavage of NOTCH receptor by the gamma secretase complex. Different inhibitors of gamma secretase have been developed (see ref. [18] for a list). These inhibitors have been tested *in vitro* on a variety of cell lines, including melanoma [12], [13], [18], [25]. Clinical data have been supplied mostly by trials in adult T Cell leukemia (ALL), but efficacy has been hindered by significant gastrointestinal toxicities associated with treatment [26]. However, RO4929097 is a novel gamma secretase inhibitor with an improved clinical toxicity profile [27]. Here, we report the preclinical effects of RO4929097 on both primary and metastatic melanoma cells. In particular, we show for the first time that the inhibition of NOTCH signaling has an impact on the tumor initiating properties of melanoma cells.

## Results

### RO4929097 affects the oncogenic and stem cell-like properties of primary melanoma cells *in vitro*

In order to evaluate the potential use of RO4929097 in the adjuvant setting, the drug was initially tested on a panel of primary melanoma cell lines. WM35, WM98.1, WM115, WM983A and WM3248 were chosen because of their aggressive phenotype, as indicated by their ability to form colonies in soft agar and to organize in spheres (Figure S1), which are nonadherent 3D structures enriched in melanoma initiating cells and characterized by increased differentiation capacity *in vitro* and tumorigenic potential *in vivo* [28], [29].

**Figure 1:**
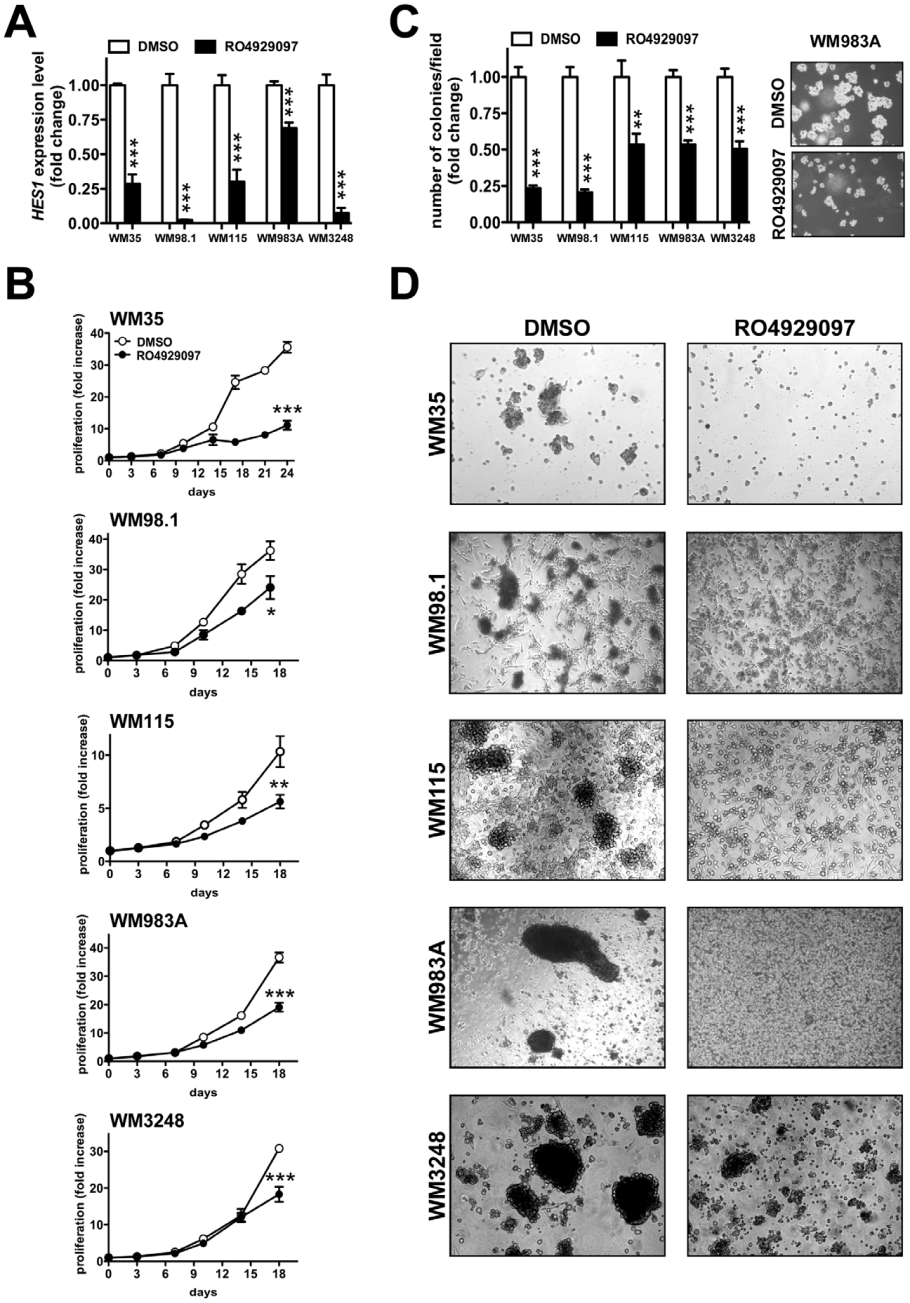
RO4929097 inhibits proliferation, anchorage independent growth, and sphere formation of primary melanoma cells *in vitro*. (**A**) RO4929097 causes a decrease in the levels of NOTCH downstream target HES1. WM35, WM98.1, WM115, WM983A and WM3248 cells were treated with DMSO (white bars) or 10 uM RO492907 (black bars) for 24 h. At that time, RNA was collected and *HES1* levels were measured by qRT-PCR. The mean±s.d. of 3 independent experiments is reported. (**B**) RO4929097 inhibits cell proliferation. The indicated cell lines were treated with DMSO (white circles) or 10 uM RO4929097 (black circles). A representative curve of three independent experiments is reported. (**C**) RO4929097 inhibits anchorage independent growth. White bars: DMSO treated cells; black bars: RO4929097 treated cells. The mean±s.d. of three independent experiments is reported. Right panels show representative images of WM983A cells. (**D**) RO4929097 impairs the formation of melanospheres. Representative pictures of one among 3 independent experiments are shown. T test, *p<0.05; **p<0.005; ***p<0.001.

Upon RO4929097 treatment, the selected melanoma cell lines showed downregulation of NOTCH downstream effector HES1 (Figure 1A), confirming the ability of the drug to affect the NOTCH signaling pathway. The impairment of NOTCH signaling was associated with a significant reduction in cell proliferation (Figure 1B) and in anchorage independent growth (Figure 1C). We then tested the ability of RO4929097 to impair the formation of melanospheres. We indeed found a decrease in the amount of melanospheres formed upon RO4929097 treatment in primary melanoma cell lines (Figure 1D).

Taken together these results suggest that RO4929097 is able to affect the oncogenic and stem cell-like properties of melanoma cells *in vitro*.

### RO4929097 impairs the growth of primary melanoma cells *in vivo*

**Figure 2:**
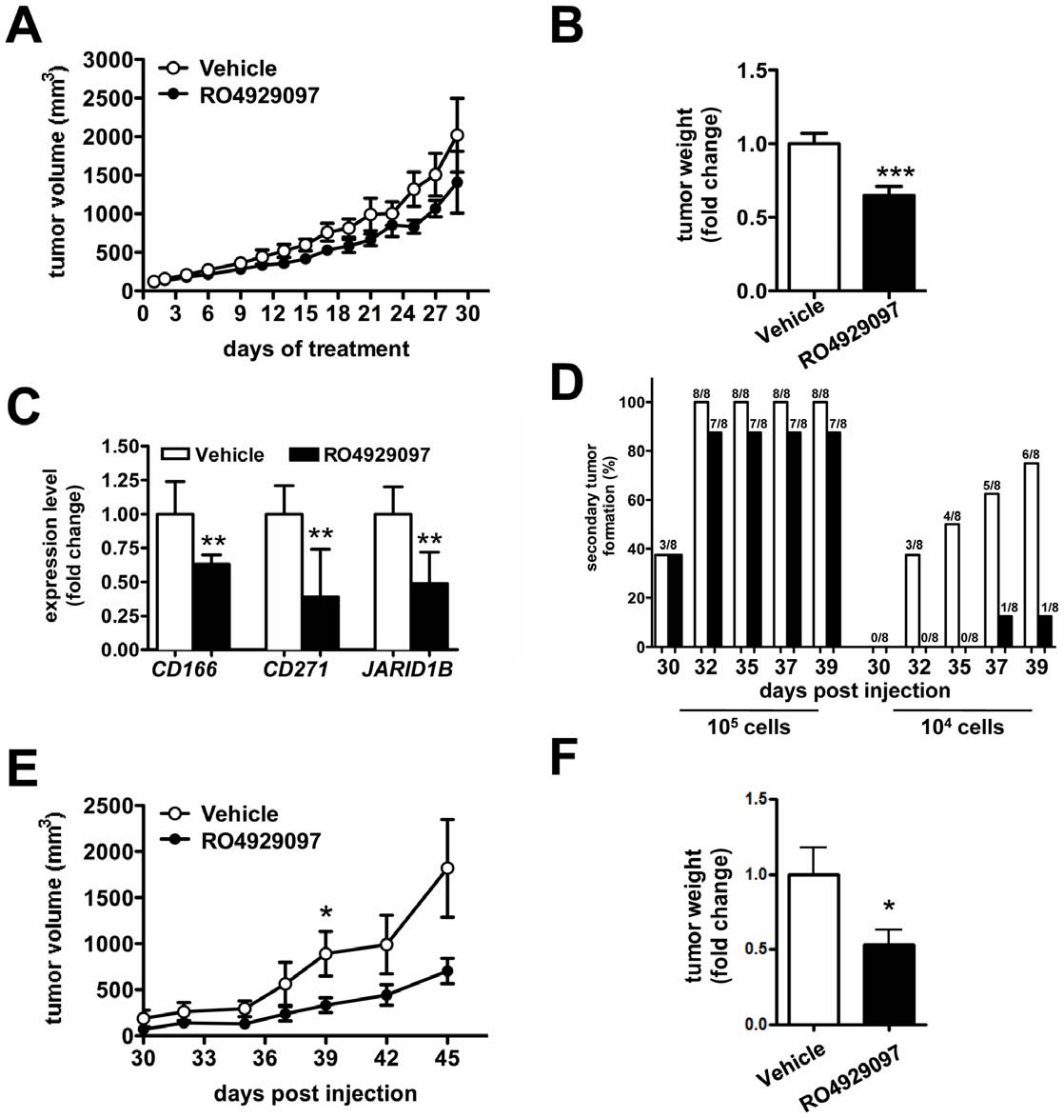
RO4929097 impairs the growth of primary melanoma cells *in vivo*. (**A–C**) Primary tumor formation of vehicle- and RO4929097-treated WM3248 cells. (**A**) 5×10^6^ WM3248 primary melanoma cells were injected in the flank of NOG mice (n = 20). Once the tumors became measurable, mice were randomized in two groups and vehicle (n = 10) or RO4929097 (n = 10) was administered orally at 10 mg/Kg/day for 30 days. Tumor volume was measured every 2–3 days. White circles: vehicle-treated mice; black circles: RO4929097-treated mice. (**B**) At the end of the treatment period, tumors were excised and weighed. White bar: vehicle-treated mice; black bar: RO4929097-treated mice. (**C**) Levels of melanoma stem cell markers *CD166*, *CD271* and *JARID1B* in WM3248 xenografts measured by qRT-PCR. White bars: vehicle-treated tumors; black bars: RO4929097-treated tumors. (**D–F**) Secondary tumor formation of vehicle and RO4929097-treated WM3248 cells. (**D**) White bars: secondary tumors formed by vehicle-treated primary tumors; black bars: secondary tumors formed by RO4929097-treated primary tumors. (**E**) Volume and (**F**) weight of the secondary tumors formed by 10^5^ WM3248 cells previously treated with vehicle (white circles/bar) or RO4929097 (black circles/bar). Tumor volume was measured every 2–3 days starting at 30 days post injection. At 45 days after the injection, tumors were excised and the weight was measured. T test, *p<0.05; **p<0.005; ***p<0.001.

To further validate the effects of NOTCH pathway inhibition, we investigated the effects of RO4929097 on the growth of the primary melanoma cell line WM3248 in NOD/SCID/IL2gammaR-/- (NOG) mice. Once measurable tumors were established, we randomly distributed the mice into groups to receive either vehicle control or RO4929097 by daily oral administration (Figure S2A). In accordance with our *in vitro* data, we found a decrease in tumor growth with RO4929097 treatment, which was more appreciable after tumors were extracted for weight assessment (Figure 2A, B). RO4929097-treated tumors also displayed lower expression of putative melanoma stem cell markers *CD166*, *CD271* and *JARID1B* [19], [21], [30] compared to vehicle-treated ones (Figure 2C). In order to formally prove that the decrease in these markers is associated with a diminished tumor-initiating ability, we used a serial xenotransplantation assay [31]. We resected primary tumors from RO4929097- or vehicle-treated mice and dissociated the cells for re-implantation into NOG mice. We compared two different cellular concentrations, 10^5^ and 10^4^ cells per flank. Secondary tumors did not receive any further treatment with either vehicle or RO4929097, while their intrinsic growth properties were monitored (Figure S2A). At the concentration of 10^5^ injected cells per flank, we did not find a significant difference between vehicle- and RO4929097-treated cells in time to secondary tumor formation. However, the percentage of secondary tumors formed by RO4929097-treated cells was lower (Figure 2D, *left*). Furthermore, the secondary tumors formed by RO4929097-treated cells were smaller (Figure 2E, F). Strikingly, a significant delay in tumor formation by the RO4929097-treated cells compared to the vehicle-treated ones was observed in mice injected with 10^4^ cells (Figure 2D, *right*). Nearly 40 days after implantation, only 1/8 flanks injected with RO4929097-treated cells had developed measurable tumors as compared to 6/8 injected with vehicle-treated cells. All together these results indicate that RO4929097 is able to affect the tumorigenic potential of melanoma cells *in vivo*.

### RO4929097 impairs the growth of metastatic melanoma cells *in vivo*

**Figure 3:**
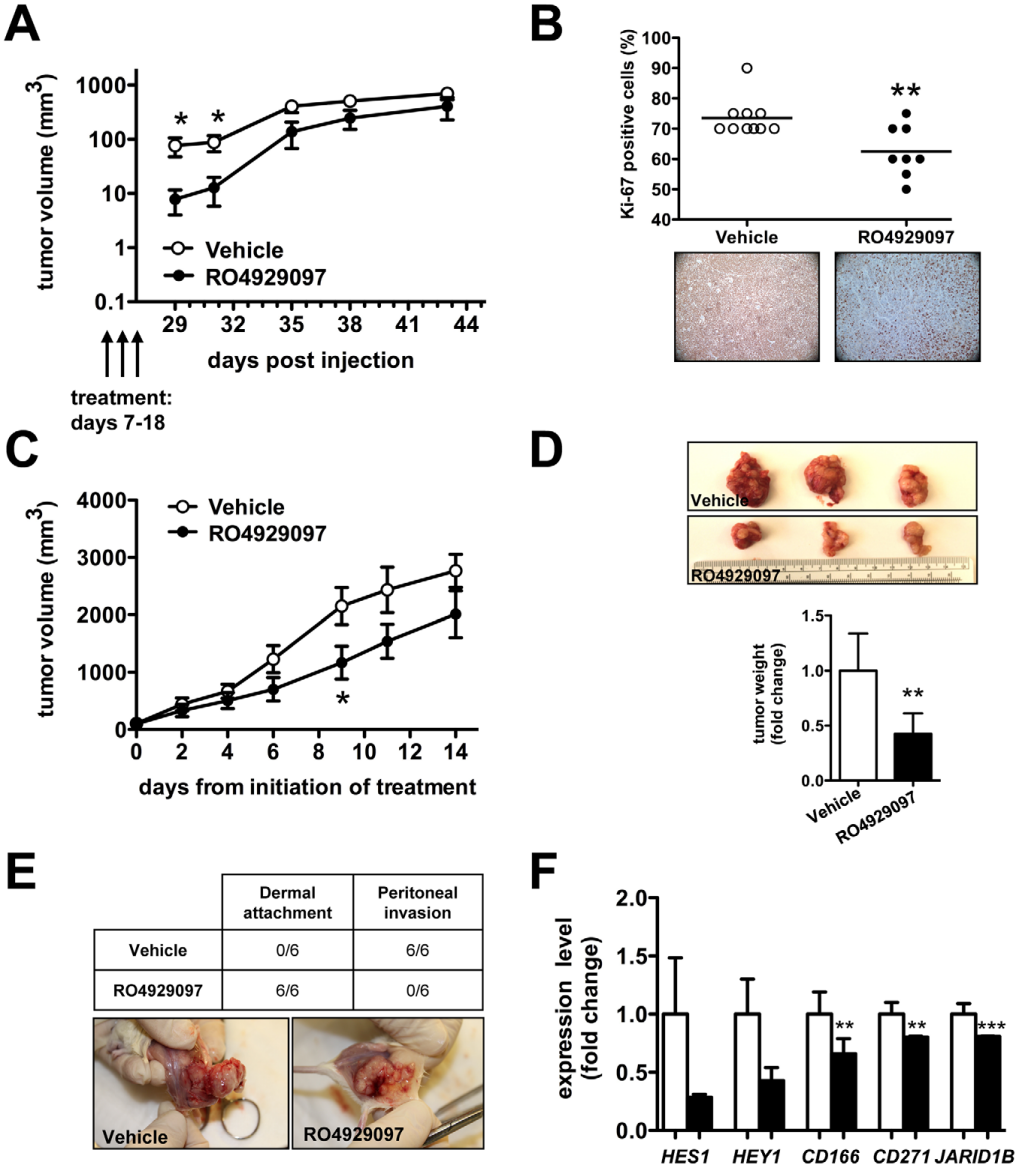
RO4929097 impairs the growth of metastatic melanoma cells *in vivo*. (**A–B**) 10^6^ 5B1 metastatic melanoma cells were injected in the flank of NOG mice (n = 20). Before tumors became measurable, mice were randomized in two groups and vehicle (n = 10) or RO4929097 (n = 10) was administered orally at 10 mg/Kg/day for 12 days. (**A**) Tumor volume, measured every 2–3 days starting 3 days after the end of the treatment. White circles: vehicle-treated mice; black circles: RO4929097-treated mice. (**B**) Percentage of Ki-67-positive cells in vehicle-treated (white circles) and RO4929097-treated (black circles) tumors. Immunohistochemistry was performed on tumor excised twenty-five days after the end of the treatment. (**C–F**) 2×10^6^ A375 metastatic melanoma cells were injected in the flanks of NOG mice (n = 12). As soon as tumors became measurable, mice were randomized in two groups, and vehicle (n = 6) or RO4929097 (n = 6) was administered orally at 10 mg/Kg/day for 2 weeks. (**C**) Tumor volume, measured every 2–3 days during the two weeks of treatment. White circles: vehicle-treated mice; black circles: RO4929097-treated mice. (**D**) Tumor weight, measured when the tumors were excised at the end of the 2 weeks of treatment. White bar: vehicle-treated mice; black bar: RO4929097-treated mice. (**E**) Invasive features (dermal attachment versus peritoneal invasion) of vehicle-treated and RO4929097-treated tumors. (**F**) qRT-PCR analysis of NOTCH downstream effectors *HES1* and *HEY1* and of melanoma stem cell markers *CD166*, *CD271* and *JARID1B* in vehicle-treated (white bars) and RO4929097-treated (black bars) tumors. T test, *p<0.05; **p<0.005; ***p<0.001.

We also tested the effects of RO4929097 on metastatic melanoma cell lines using two *in vivo* xenograft models. We first assessed the impact of RO4929097 on tumor onset by treating NOG mice with the compound for 12 days, starting 7 days after flank injection of 5B1 melanoma cells (Figure S2B). In this experimental setting, we found a significant delay in tumor formation in RO4929097-treated mice compared to vehicle treated ones (Figure 3A). RO4929097-treated tumors were characterized by reduced proliferative index, as revealed by Ki-67 staining (Figure 3B). We then assessed the impact of RO4929097 treatment on the growth of pre-existing tumors, by initiating the treatment only after measurable tumors were established (Figure S2C). RO4929097 treatment negatively affected the volume (Figure 3C) and especially the weight (Figure 3D) of A375 tumors xenografted into NOG mice, without increasing the number of apoptotic cells (Caspase 3 staining on resected tumors, not shown). A different *in vivo* growth pattern associated with RO4929097 treatment was also observed: compound-treated tumors grew along the subcutaneous dermal borders, as opposed to vehicle-treated tumors that consistently invaded the peritoneum (Figure 3E). The expression of the NOTCH targets HES1 and HEY1 was reduced in RO4929097-treated tumors, together with that of putative melanoma stem cell markers (Figure 3F). Previous studies have shown significant toxicity, particularly secretory diarrhea, associated with gamma secretase inhibition. In contrast, we did not observe any significant weight changes or overt abnormalities in the organs of RO4929097-treated mice compared to vehicle-treated ones (Figure S3).

### NOTCH activity predicts melanoma patient outcome

**Figure 4:**
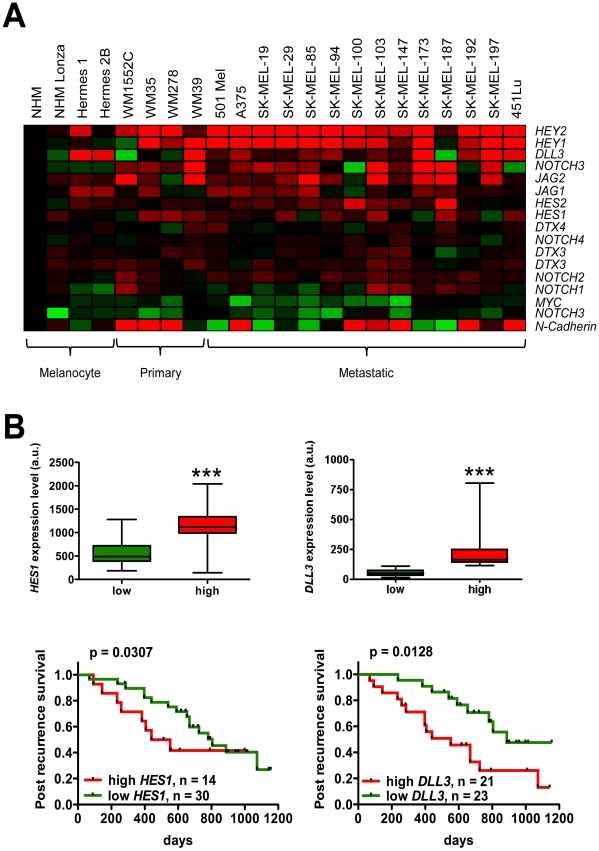
NOTCH pathway is upregulated in human melanoma metastasis and correlates with worse prognosis. (**A**) mRNA array analysis of NOTCH pathway members. Affymetrix U133A 2.0 array performed on 4 melanocyte controls (2 normal human melanocytes and 2 immortalized melanocytes), 4 primary and 14 metastatic cell lines [34] reveals the upregulation of NOTCH pathway members, such as *JAG1*, *JAG2*, *DLL3*, *NOTCH3*, *HES1*, *HES2*, *HEY1* and *HEY2* in melanoma. (**B**) Post recurrence survival of melanoma patients showing low and high levels of NOTCH pathway members *HES1* (left) and *DLL3* (right). Previously published gene expression data of 44 metastatic melanoma tissue samples [37] were used to define “low” and “high” expressor groups (upper panels, Wilcoxon test, ***p<0.001) and to generate Kaplan-Meier curves (lower panels, log-rank test).

The analysis of gene expression arrays recently published by our group and others [32], [33], [34] shows that several NOTCH signaling pathway components are overexpressed in primary as well as in metastatic melanoma compared to melanocytic controls (Figure 4A and Figure S4), confirming the notion that NOTCH signaling pathway is upregulated in melanoma [35], [36]. However, to our knowledge, a potential association between aberrant NOTCH signaling and prognosis in melanoma patients has not been shown. Using the Affymetrix expression profile of 44 metastatic melanoma samples from patients followed clinically for a median of 20 months (2–38 months range) [37], we examined whether NOTCH signaling components can be prognostic markers in metastatic melanoma. We indeed found that higher *HES1* and *DLL3* levels were significantly associated with decreased post recurrence survival as continuous predictors in Cox regression analysis (Figure 4B).

## Discussion

The aggressiveness of melanoma, which is surprisingly high considering that it is among a handful of cancers whose dimensions are reported in millimeters, is due to the high degree of heterogeneity and plasticity combined with the chemoresistance of melanoma cells [38], [39], [40]. Therefore, new treatment strategies that selectively target the most resistant cells within the tumors, potentially the stem cell-like melanoma initiating cells, are urgently needed [41], [42].

RO4929097 has been previously shown to be a potent and selective gamma secretase inhibitor with promising antitumoral activity *in vivo* and without the toxicity associated with other gamma secretase inhibitors [27]. Here, RO4929097 was shown for the first time to affect the tumor initiating ability of melanoma cells. We found that RO4929097 suppresses the oncogenic properties of several primary melanoma cell lines, as indicated by the decrease in proliferation and in the number of colonies formed in soft agar by RO4929097-treated cells. More importantly, we found that RO4929097 impairs the formation of melanospheres (Figure 1). In order to confirm *in vivo* that RO4929097 can affect the tumor initiating ability of melanoma cells, we used the serial xenotransplantation assay. This assay is the gold standard in assessing self-renewal, which is the defining property of cancer stem cells [31]. Strikingly, the serial xenotransplantation assay showed a significant delay in tumor formation by the RO4929097-treated cells compared to the vehicle-treated ones (Figure 2). The decrease in size and incidence and the increase in latency of the secondary tumors formed by RO4929097-treated cells are particularly remarkable since we did not sort these cells for any specific marker, such as CD271 and ABCB5 [19], [20]. Individual markers of tumor initiating cells have been challenged by the experimental evidence that most melanoma cells harbor tumorigenic capacity, irrespective of their phenotypic characteristics [22]. Our data indicating that RO4929097 can affect the tumorigenicity of melanoma cells further support the inhibition of NOTCH signaling as a promising therapeutic strategy for the eradication of melanoma, as suggested for breast cancer and glioma [43], [44], [45].

Although it is important to understand alterations essential to primary transformation, the clinical obstacle in melanoma treatment is the dearth of effective therapeutics in the metastatic setting. In addition to its role in melanomagenesis, aberrant NOTCH signaling has been shown to promote metastasis in melanoma and other cancers [12], [46]. Due to all these findings, we decided to test the efficacy of RO4929097 *in vivo* on metastatic melanoma cell lines. We observed that RO4929097 not only interferes with the ability of metastatic melanoma cells to form tumors when injected into nude mice, but also impairs the growth of pre-existing tumors (Figure 3). All together, these data lend further support to the notion that NOTCH signaling can play a role in advanced melanoma and offer a rationale to explore the therapeutic potential of NOTCH inhibition in the metastatic setting.

Finally, the analysis of gene expression arrays recently published by our group and others showed that several NOTCH signaling pathway components are overexpressed in metastatic cell lines in comparison to melanocytes. Furthermore, we report for the first time that aberrant NOTCH signaling can predict clinical outcome in melanoma, with high *HES1* and *DLL3* levels associated with shorter post recurrence survival (Figure 4). These results are in agreement with data previously reported in other tumor types, such as breast cancer [47], [48] and neuroblastoma [49].

Molecular classification of melanoma is an emerging theme in melanoma therapeutics, with BRAF and c-KIT mutation status determining novel treatment options [50], [51]. Although RO4929097 shows antitumor activity in both primary and metastatic cell lines, response to RO4929097 is not universal. Not all melanoma cell lines are (equally) sensitive in all the assays, as exemplified by the fact that some of the melanoma cell lines we tested were not responsive to the compound (data not shown) and by the different degree of reduction in proliferation and colony formation in soft agar showed by the 5 responsive cell lines reported in Figure 1. This differential sensitivity may be dependent on a unique molecular signature. The signature of responders to RO4929097 will emerge as clinical data accumulate from ongoing trials with this compound in melanoma, and will help to define the subset of patients that will benefit the most from RO4929097 treatment. These studies may also help to conceive combinatorial treatments of RO4929097, which, even when effective, does not cause a profound inhibition of tumor growth (Figure 1B, 2A, 3A, 3C), but is able to affect tumor initiating ability (Figure 2D), with other drugs that, conversely, are more effective at preventing tumor growth, but fail to decrease the tumor initiating ability. Alternatively, RO4929097 could also be effective in the adjuvant setting to prevent metastatic spread.

In summary, our preclinical studies support the gamma secretase inhibition as a novel approach that is able to target the melanoma initiating pool and offer insights into the clinical potential of this new treatment strategy.

## Materials and Methods

### Human melanoma cell lines

Primary melanoma cell lines WM35, WM98.1, WM115, WM983A and WM3248 were purchased from the Wistar Institute (Philadelphia, PA) and cultured in Mel 2% medium [28]. A375 metastatic melanoma cell line was purchased from ATCC and cultured in DMEM +10% FBS. WM239A/131/4-5B1 (5B1) metastatic melanoma cell line, a kind gift from Robert S. Kerbel (University of Toronto), was cultured in DMEM+10% FBS.

### Quantitative real time PCR (qRT-PCR)

Total RNA was extracted using Trizol reagent according to the manufacturer's instructions. It was then subjected to DNase treatment and retrotranscription (1ug RNA in a 20 ul reaction). Real-time PCR of *HES1*, *HEY1*, *CD166*, *CD271*, and *JARID1B* was performed using Sybr green fluorescence. 2 ul of RT were used in a 20 ul reaction. *GAPDH* was used as an internal standard. Relative quantification of gene expression was performed with the comparative CT method [52]. The sequences of the primers used are described in Table S1.

### Proliferation assay

Cells were seeded at 2.5×10^3^ cells per well on a 12-well dish in triplicate. The day after (day 0), the medium was replaced, and DMSO or 10 uM RO4929097 was added and changed every 3–4 days. At the indicated time points, cells were fixed in 10% formalin solution and stored in PBS at 4°C. At day 18–24, all the plates were stained with crystal violet. After color elution with 10% acetic acid, optical density was read at 590 nm. A representative curve of three independent experiments is reported.

### Growth in semisolid medium

The bottom layer was obtained by covering 6-well dishes with 3 ml of 0.6% agar in MCDB 153 medium [28]. The day after, 5×10^4^ cells pre-treated for 24 h with 10 uM RO4929097 or vehicle were seeded in triplicate in 2 ml Mel 2% medium containing 0.3% agar and 10 uM RO4929097 or vehicle. Colonies were counted after 3–4 weeks.

### Melanosphere formation assay

Cells were seeded at a density of 5×10^5^ per 6 cm dish in Mel 2% medium. The day after, the medium was switched to ES cell medium (hESCM4, Invitrogen) containing either DMSO or 10 uM RO4929097. The medium was left unchanged for two weeks, and then half of the medium was changed once a week, refreshing the drug. Spheres were visible in the DMSO treated dishes after 6–12 weeks.

### Serial xenotransplantation assay of WM3248 cells

5×10^6^ WM3248 primary melanoma cells were injected in the flank of NOG mice (n = 20). Once the tumors became measurable, mice were randomized in two groups and vehicle (n = 10) or RO4929097 (n = 10) was administered orally at 10 mg/Kg/day according to the protocol described in [27]. Tumor volume was measured every 2–3 days for a total of 30 days (Figure S2A). At the end of the treatment period, tumors were excised and weighed. A piece of the excised tumors was cut and used for RNA extraction and qRT-PCR. The rest of the tumor was mechanically dissociated and passed through 100 um and 40 um strainers (BD Falcon). Viable cells were counted and 10^5^ and 10^4^ cells were re-injected in the flanks of NOG mice (n = 8) without any further treatment with RO4929097. At the indicated time points, the presence of tumor was checked. Tumor volume was measured every 2–3 days starting at 30 days post injection. At 45 days after the injection, tumors were excised and the weight was measured.

### Xenograft assay of 5B1 and A375 cells

10^6^ 5B1 cells were injected in the flank of NOG mice (n = 20). Before tumors became measurable, mice were randomized in two groups and vehicle (n = 10) or RO4929097 (n = 10) was administered orally at 10 mg/Kg/day for 12 days (Figure S2B). Twenty-five days after the end of the treatment, tumor were excised, formalin fixed and paraffin embedded.

2×10^6^ A375 cells were injected in the flanks of NOG mice (n = 12). As soon as tumors became measurable (60–100 mm^3^), mice were randomized in two groups, and vehicle (n = 6) or RO4929097 (n = 6) was administered orally at 10 mg/Kg/day for 2 weeks (Figure S2C). Tumor volume was measured every 2–3 days during the two weeks of treatment. Tumor weight was measured when the tumors were excised at the end of the 2 weeks of treatment.

### Ethics statement

Experiments were conducted following protocols approved by the NYU Institutional Animal Care Use Committee (IACUC) (protocol number 080109).

### Immunohistochemistry (IHC)

Ki-67 IHC was performed using mouse anti-human Ki-67 monoclonal antibody (Neomarkers/Lab Vision, Fremont, CA, USA). Sections were deparaffinized in xylene (3 changes), rehydrated through graded alcohols (3 changes 100% ethanol, 3 changes 95% ethanol) and rinsed in distilled water. Heat-induced epitope retrieval was performed in a 1200-Watt microwave oven at 100% power in 10 mM citrate buffer pH 6.0 for 20 min. Sections were allowed to cool down for 30 min and then rinsed in distilled water. Antibody incubation and detection were carried out at 37°C on a NEXes instrument (Ventana Medical Systems Tucson, Arizona) using Ventana's reagent buffer and detection kits. Endogenous peroxidase activity was blocked with hydrogen peroxide. Ki-67 was diluted 1∶400 and incubated for 30 min at room temperature. Biotinylated goat anti-mouse followed by application of streptavidin-horseradish-peroxidase conjugate was used to detect the primary antibody. The complex was visualized with 3,3-diaminobenzidene and enhanced with copper sulfate. Slides were washed in distilled water, counterstained with hematoxylin, dehydrated and mounted with permanent media. Appropriate positive controls were included with the study sections. Blinded to mouse groups, an attending pathologist (F.D.) scored the percentage of Ki-67-positive cells in each slide.

### Analysis of previously published datasets

Gene expression data of 44 metastatic melanoma tissue samples previously published by our group [37] were used to define “low” and “high” expressor groups for HES1 and DLL3 (Wilcoxon test) and to generate Kaplan-Meier curves (log-rank test). The binary classification of gene expression uses a cut-off point derived using the statistical method described in [53], which is based on maximizing the absolute value of log-rank statistic. Expression values for both HES1 and DLL3 are statistically significant as continuous predictors of survival in Cox proportional hazard regression models (p values<0.03).

## Supporting Information

Figure S1 **Primary melanoma cell lines form spheres when switched to ES medium.** WM35, WM98.1, WM115, WM983A and WM3248 (left panels) organize in three-dimensional melanospheres (right panels, arrows) when switched to ES medium.
(TIF)

Figure S2 **Schemes of drug treatment and toxicity.** (**A**) Scheme of treatment for WM3248 xenograft. 5×10^6^ WM3248 primary melanoma cells were injected in the flanks of NOG mice (10 per group). Once tumors were measurable, vehicle or RO4929097 was administered orally at 10 mg/Kg/day for 30 days. At day 42, mice were sacrificed and the tumors dissected and mechanically dissociated. 10^4^ and 10^5^ cells from vehicle and compound treated tumors were injected in the flank of NOG mice (8 flanks/group) and tumor formation was followed for 45 days. (**B**) Scheme of treatment for 5B1 xenograft. 10^6^ 5B1 metastatic melanoma cells were injected in the flank of NOG mice (10 per group). Before tumors became measurable, vehicle or RO4929097 was administered orally at 10 mg/Kg/day for 12 days. Treatment was stopped and tumor volume was started to be measured. (**C**) Scheme of treatment for A375 xenograft. 2×10^6^ A375 metastatic melanoma cells were injected in the flank of NOG mice (6 per group). After the tumor became measurable, vehicle or RO4929097 was administered orally at 10 mg/Kg/day for 2 weeks.
(TIF)

Figure S3 **Toxicity of RO4929097.** Throughout the treatment period, mice treated with RO4929097 (black circles) did not show any weight loss compared with the vehicle treated ones (white circles).
(TIF)

Figure S4 **Expression of Notch related genes in previously published data sets.** (**A**) mRNA expression of the NOTCH ligand DLL3 (left) and the NOTCH target gene HEY1 (right). One-way variance ANOVA test was applied. (**B**) mRNA expression of the ligand DLL3 (up, left) and the targets HEY1 (up, right), HEY2 (down, left) and N-Cadherin (down, right). Unpaired t test with Welch's correction was applied. NHEM indicates the expression in the melanocytic lineage.
(TIF)

Table S1 **Real time PCR primers.** List of primer pairs used to amplify the indicated mRNAs by quantitative PCR.
(TIF)
