# Correction: The Novel Gamma Secretase Inhibitor RO4929097 Reduces the Tumor Initiating Potential of Melanoma

**DOI:** 10.1371/annotation/a89c089f-ae9d-4453-a314-37efd5efb126

**Published:** 2011-11-02

**Authors:** Chanh Huynh, Laura Poliseno, Miguel F. Segura, Ratna Medicherla, Adele Haimovic, Silvia Menendez, Shulian Shang, Anna Pavlick, Yongzhao Shao, Farbod Darvishian, John F. Boylan, Iman Osman, Eva Hernando

The authors wish to add the following to their Funding section: "This initiative is funded by the CTSI (grant by number 1 UL1 RR029893)." 

