# Supplementary figures and images for: The Novel Gamma Secretase Inhibitor RO4929097 Reduces the Tumor Initiating Potential of Melanoma
Source: PLoS One. 2011 Sep 29;6(9):e25264. doi: 10.1371/journal.pone.0025264 (PMC3182998; doi:10.1371/journal.pone.0025264)

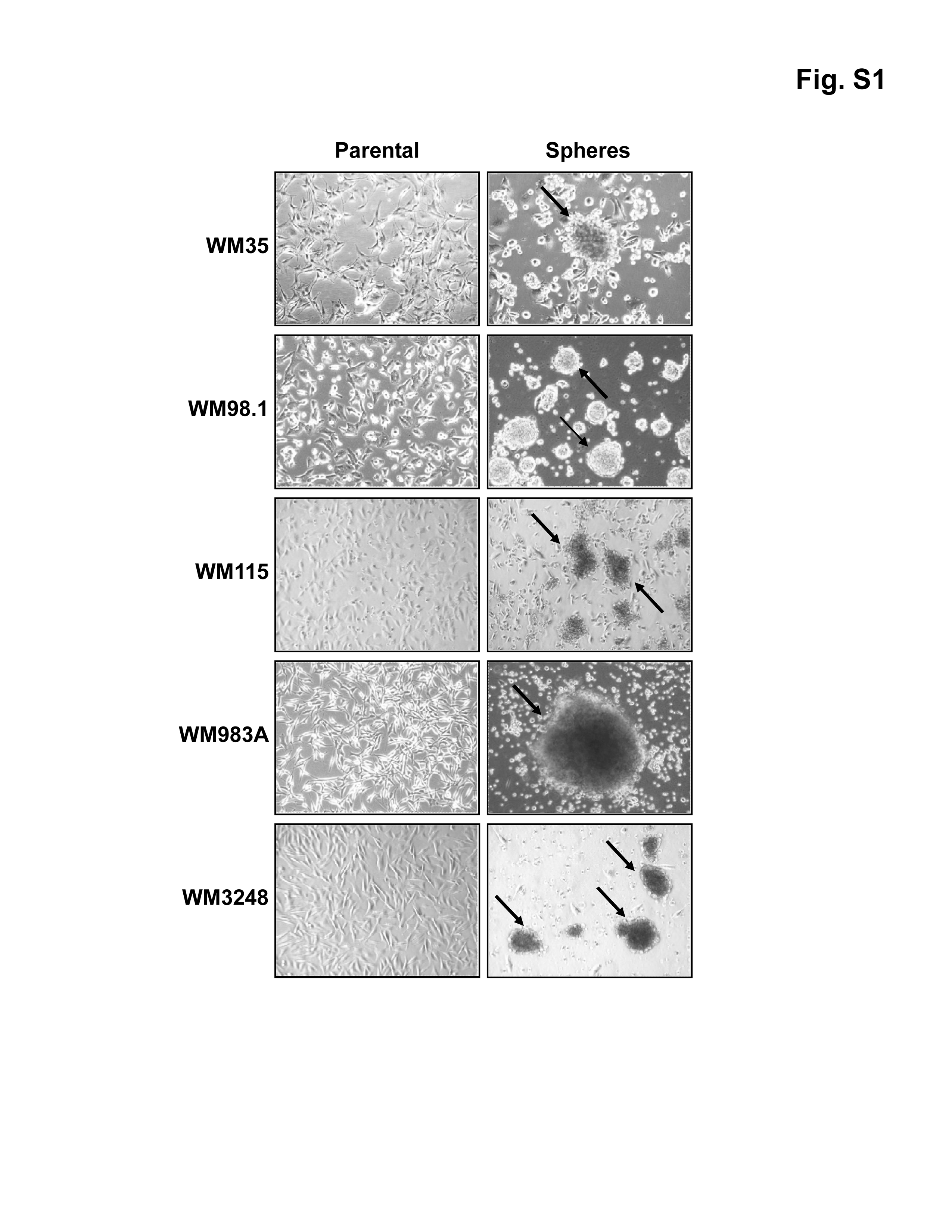

Supplement: Figure S1 — Primary melanoma cell lines form spheres when switched to ES medium. WM35, WM98.1, WM115, WM983A and WM3248 (left panels) organize in three-dimensional melanospheres (right panels, arrows) when switched to ES medium. (TIF) [file pone.0025264.s001.tif]

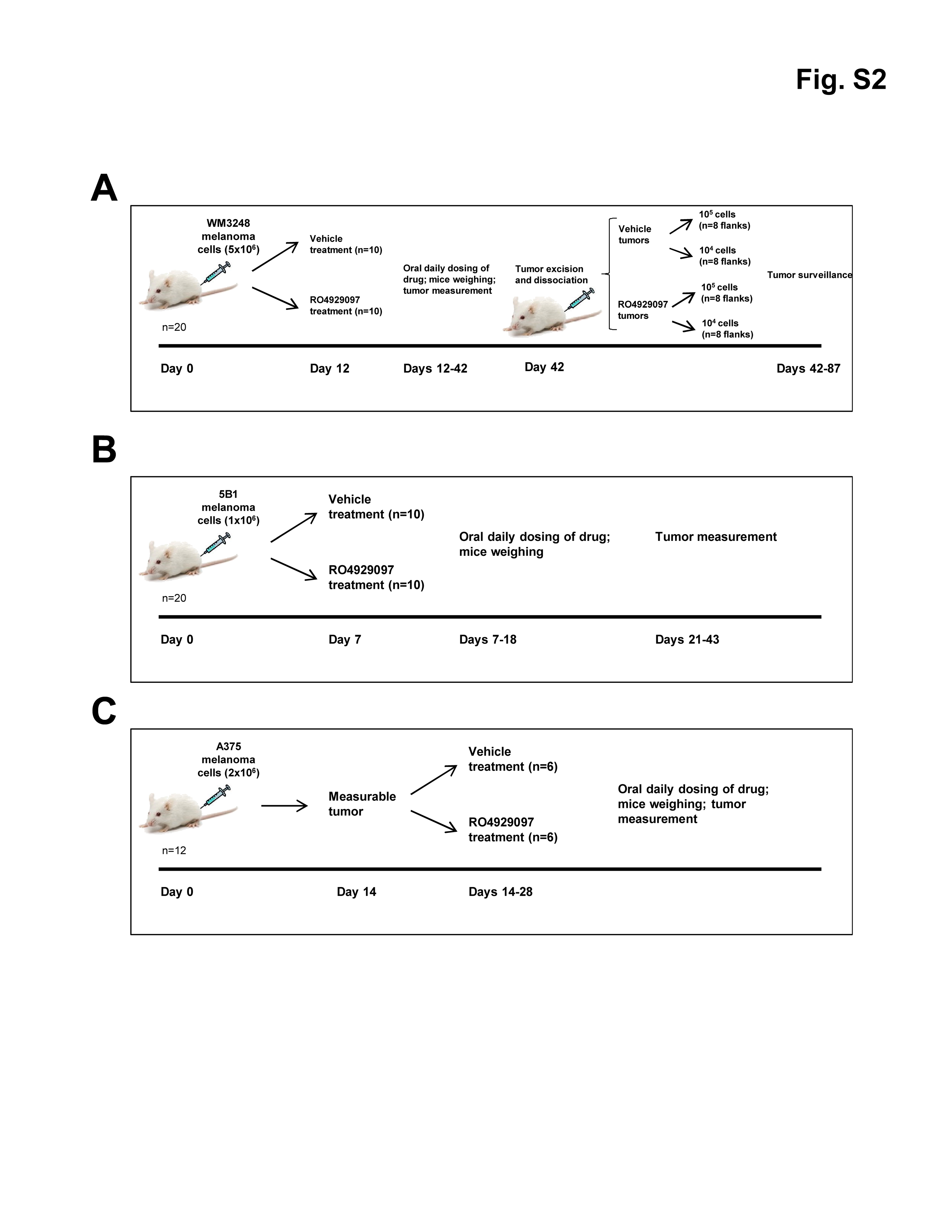

Supplement: Figure S2 — Schemes of drug treatment and toxicity. (A) Scheme of treatment for WM3248 xenograft. 5×10^6^ WM3248 primary melanoma cells were injected in the flanks of NOG mice (10 per group). Once tumors were measurable, vehicle or RO4929097 was administered orally at 10 mg/Kg/day for 30 days. At day 42, mice were sacrificed and the tumors dissected and mechanically dissociated. 104 and 105 cells from vehicle and compound treated tumors were injected in the flank of NOG mice (8 flanks/group) and tumor formation was followed for 45 days. (B) Scheme of treatment for 5B1 xenograft. 106 5B1 metastatic melanoma cells were injected in the flank of NOG mice (10 per group). Before tumors became measurable, vehicle or RO4929097 was administered orally at 10 mg/Kg/day for 12 days. Treatment was stopped and tumor volume was started to be measured. (C) Scheme of treatment for A375 xenograft. 2×10^6^ A375 metastatic melanoma cells were injected in the flank of NOG mice (6 per group). After the tumor became measurable, vehicle or RO4929097 was administered orally at 10 mg/Kg/day for 2 weeks. (TIF) [file pone.0025264.s002.tif]

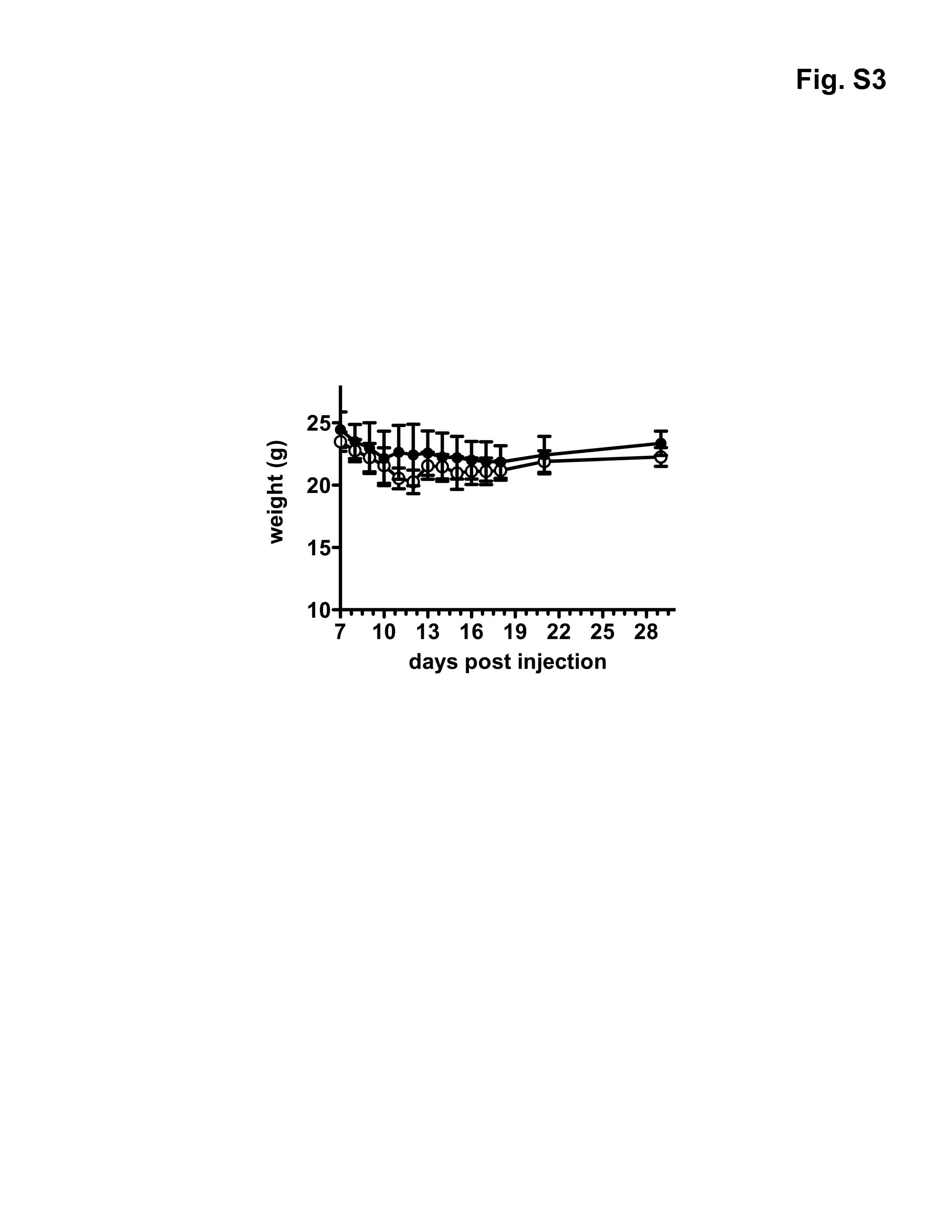

Supplement: Figure S3 — Toxicity of RO4929097. Throughout the treatment period, mice treated with RO4929097 (black circles) did not show any weight loss compared with the vehicle treated ones (white circles). (TIF) [file pone.0025264.s003.tif]

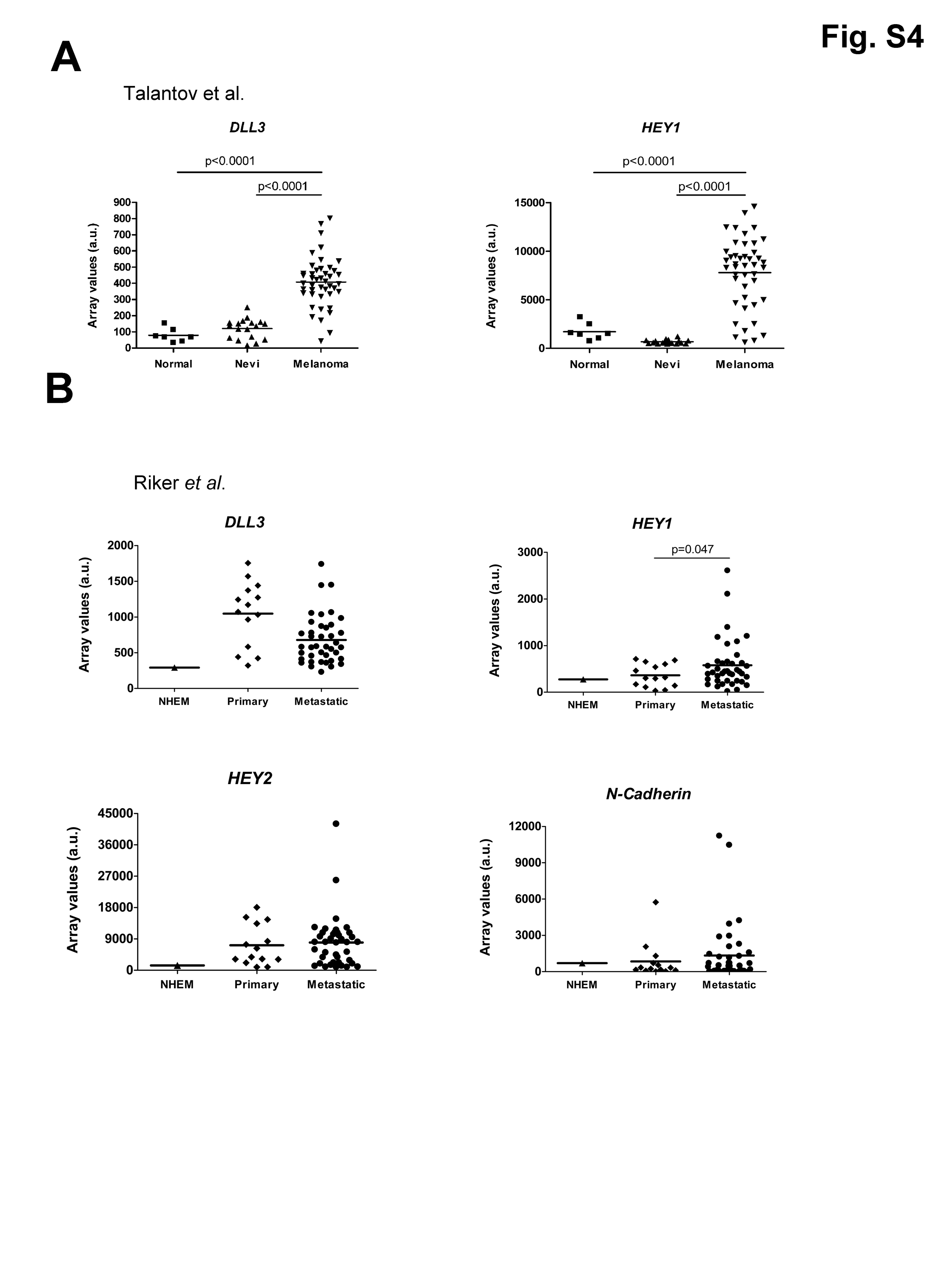

Supplement: Figure S4 — Expression of Notch related genes in previously published data sets. (A) mRNA expression of the NOTCH ligand DLL3 (left) and the NOTCH target gene HEY1 (right). One-way variance ANOVA test was applied. (B) mRNA expression of the ligand DLL3 (up, left) and the targets HEY1 (up, right), HEY2 (down, left) and N-Cadherin (down, right). Unpaired t test with Welch's correction was applied. NHEM indicates the expression in the melanocytic lineage. (TIF) [file pone.0025264.s004.tif]
